# Probability analysis of hepatocellular carcinoma in hepatitis patients in the gray zone

**DOI:** 10.3389/fmed.2024.1464981

**Published:** 2024-12-24

**Authors:** Jianna Zhang, Sijie Yu, Kailu Zhu, Shibo Li, Yu Huang

**Affiliations:** ^1^Department of Nephrology, The First Affiliated Hospital of Wenzhou Medical University, Wenzhou, China; ^2^Department of Infectious Diseases, Zhoushan Hospital, Wenzhou Medical University, Zhoushan, China; ^3^Department of Infectious Diseases, Taizhou First People's Hospital, Taizhou, China; ^4^Department of Infectious Diseases, The First Affiliated Hospital of Wenzhou Medical University, Wenzhou, China

**Keywords:** gray zone, hepatocellular carcinoma, risk probability, IPTW, chronic hepatitis B

## Abstract

**Objective:**

To investigate the probability of hepatocellular carcinoma (HCC) in a large number of gray-zone (GZ) patients with chronic hepatitis B (CHB) in clinical practice.

**Methods:**

The patients with CHB who were diagnosed and treated in our hospital from January 2013 to January 2023 were analyzed retrospectively.

**Results:**

According to the different levels of HBeAg, ALT and HBV DNA, GZ patients were divided into four categories: (1) Gray zone A (GZ-A): HBeAg positive, normal ALT level, HBV DNA ≤ 10^6^ IU/ml; (2) Gray zone B (GZ-B): HBeAg positive, ALT>ULN, HBV DNA ≤ 2 × 10^4^ IU/ml; (3) Gray zone C (GZ-C): HBeAg negative, normal ALT level, HBV DNA ≥ 2 × 10^3^ IU/ml; and (4) Gray zone D (GZ-D): HBeAg negative, ALT > ULN, serum HBV DNA ≤ 2 × 10^3^ IU/ml. This observational study showed that after adjustment using inverse probability of treatment weighting (IPTW), the probability of developing HCC in the GZ group was similar to that in the immune-tolerant, HBeAg-positive immune active, and inactive groups. The IPTW-adjusted analysis revealed that the probability of developing HCC in the GZ-B subgroup was similar to that in the immune-active and HBeAg-negative immune-active groups.

**Conclusion:**

The GZ group and GZ-B subgroup have a higher risk of HCC. Anti-hepatitis B virus therapy should be considered as early as possible for patients in the GZ group, especially in the GZ-B subgroup.

## 1 Introduction

The natural history of chronic HBV infection depends mainly on its virological, biochemical, and histological characteristics (1–3), which is generally divided into four stages (3–6) according to the status of hepatitis B e antigen (HBeAg), alanine aminotransferase (ALT) and HBV DNA levels: (1) immune-tolerant stage, (2) HBeAg-positive immune active stage, (3) inactive stage, and (4) HBeAg-negative immune active stage.

At present, many chronic hepatitis B (CHB) patients do not fit into any of these four stages based on HBeAg, ALT and HBV DNA levels, who are in the “uncertain stage” (1–3) and haven't received much attention, accounting for 28%−55% (7, 8). Patients in the “uncertain stage” are also referred to as gray zone (GZ). According to different levels of HBeAg, ALT and HBV DNA (8, 9), GZ patients are divided into four categories: (1) Gray zone A (GZ-A): HBeAg positive, normal ALT level, HBV DNA ≤

10^6^ IU/ml; (2) Gray zone B (GZ-B): HBeAg positive, ALT level above the upper limit of normal (ULN), HBV DNA ≤ 2 × 10^4^ IU/ml; (3) Gray zone C (GZ-C): HBeAg negative, normal ALT level, HBV DNA ≥ 2 × 10^3^ IU/ml; (4) Gray zone D (GZ-D): HBeAg negative, ALT > ULN, serum HBV DNA ≤ s10^3^ IU/ml. Among them, the ALT ULN is 25 U/L for females and 35 U/L for males (1, 8).

Approximately 72.7% of CHB patients in GZ have significant histological disease (SHD), namely liver inflammation ≥G2 and/or liver fibrosis ≥S2 (10). The incidence of hepatocellular carcinoma (HCC) in these patients is not yet clear. In addition, GZ patients are divided into GZ-A-GZ-D subgroups (10), with unclear risk of HCC in each subgroup. Therefore, this article focuses on the probability of developing HCC in GZ patients who have not received antiviral treatment.

## 2 Method

### 2.1 Study population

The patients with CHB who were diagnosed and treated in the First Affiliated Hospital of Wenzhou Medical University from January 2013 to January 2023 were analyzed retrospectively in this study.

All patients enrolled and followed up for 1 year in this study were CHB patients without cirrhosis who met the baseline criteria for enrollment within 1 year after baseline. Patients with metabolic associated fatty liver disease (MAFLD) during enrollment or throughout follow-up were excluded. No antiviral drugs or interferon injections were taken during enrollment and follow-up.

The diagnostic criteria for CHB is positive serum hepatitis B surface antigen (HBsAg) for more than 6 months. Patients with the following conditions were excluded: acute or chronic hepatitis C/alcoholic liver disease/MAFLD/primary biliary cholangitis/autoimmune hepatitis/hepatolenti- cular degeneration/cirrhosis/decompensation of cirrhosis/HCC/liver transplantation/AIDS/acquired immunodeficiency syndrome/other malignant tumors.

Cirrhosis is defined as the presence of any of the following conditions: ultrasound examination showing rough liver echoes or nodules on the liver surface, or CT/MRI indicating clinical features of cirrhosis and portal hypertension (e.g., ascites, splenomegaly, and varices), or thrombocytopenia (<10^5^/mm^3^) (11).

This study has been approved by the Ethics Committee of the First Affiliated Hospital of Wenzhou Medical University and was conducted in accordance with the Helsinki Declaration.

### 2.2 Data collection

Patient information such as age, gender, total bilirubin (TB), ALT, AST, γ-GT, alkaline phosphatase (ALP), albumin, platelet count (PLT), HBV-DNA, HBsAg, FIB-4, APRI, ALBI values, and presence of hypertension or diabetes was collected.

### 2.3 Statistical methods

All patients were included in the analysis based on those who still met the initial inclusion criteria from the start of enrollment to the following year, not taking into account the phase transition of patients during the follow-up period 1 year after enrollment.

In order to reduce the effects of selection bias and potential confounding factors between the two groups, differences in baseline characteristics (excluding ALT, AST, and HBV-DNA levels) were adjusted by inverse probability of treatment weighting (IPTW). The variables used for IPTW included age, gender, albumin, total bilirubin level, platelet count, diabetes, and hypertension (12, 13).

Continuous variables were expressed as the median (quartile range) and analyzed using the Mann Whitney U test. Categorical variables were analyzed using the chi square or Fisher's exact test. Statistical significance was defined as *p* < 0.05. All statistical analyses were conducted using Stata (version 17.0) and R language (R version 4.2.3).

## 3 Results

### 3.1 Characteristics of the study population

**Figure 1:**
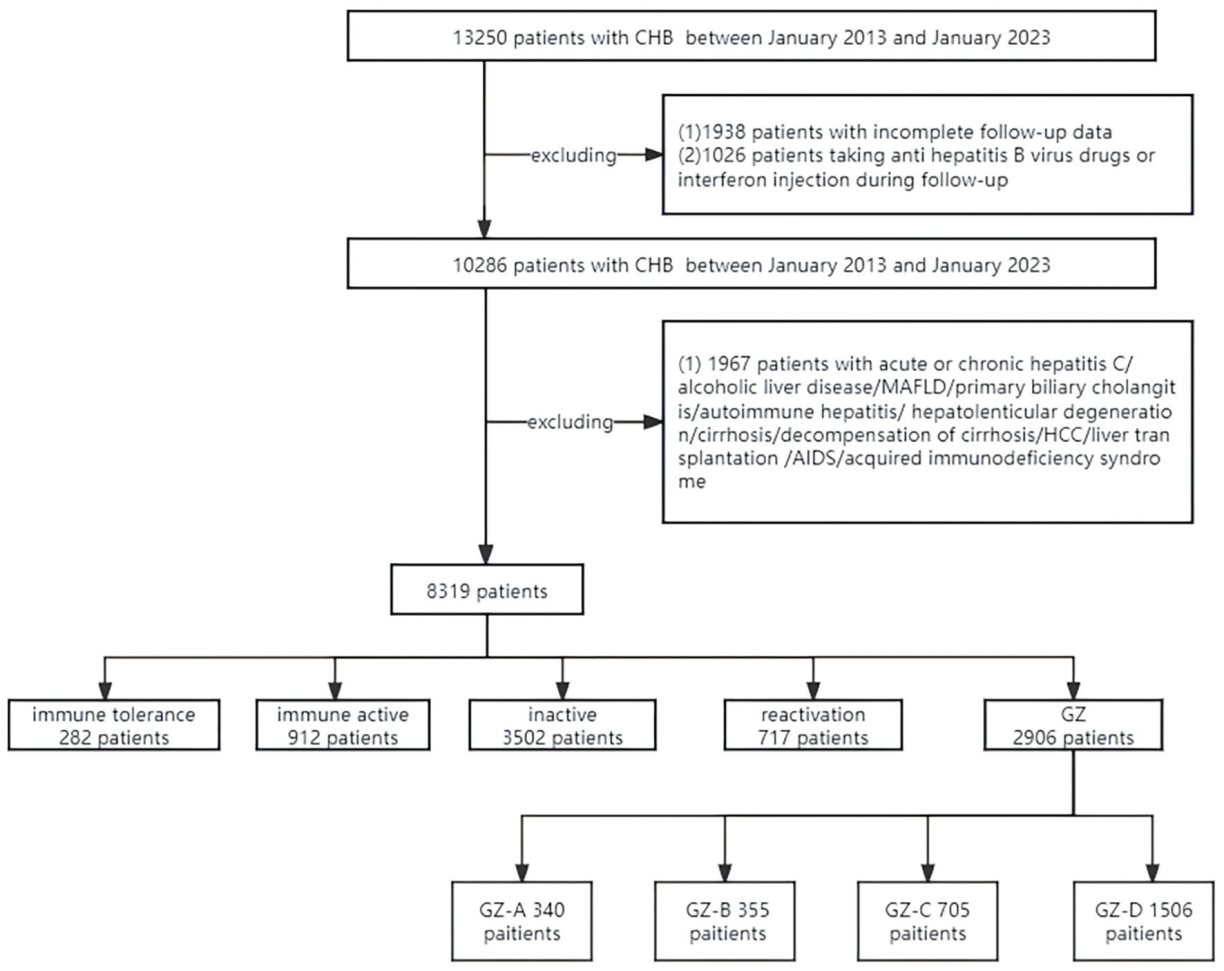
Screening process diagram for enrolled patients.

A total of 8,319 patients were involved in this study. A clinical data table was established for 5 groups of CHB patients, including the immune-tolerant, iHBeAg-positive immune -activity, inactive, HBeAg-negative immune-activation, and GZ groups, as shown in Table 1, with (1) 282 cases in the immune-tolerant group, (2) 912 cases in the HBeAg-positive immune active group, (3) 3,502 cases in the inactive group, (4) 717 cases in the HBeAg-negative immune-active group, and 2,906 cases in the GZ group (Figure 1).

**Table 1:** Baseline characteristics of patients in the immune-tolerant, HBeAg-positive immune -activity, inactive, HBeAg-negative immune-activation, and GZ groups.

**Factor**	**Immune-tolerant CHB**	**HBeAg-positive immune-active CHB**	**Inactive CHB**	**HBeAg-negative immune-active CHB**	**GZ**	***P*-value**
N	282	912	3,502	717	2,906	
Sex (male)	157 (55.7%)	606 (66.4%)	2,158 (61.6%)	517 (72.1%)	1,978 (68.1%)	<0.001
AGE, median (IQR)	34 (26, 46)	36 (28, 47)	54 (45, 64)	49 (40, 58)	50 (39, 59)	<0.001
HT						<0.001
0	260 (92.2%)	847 (92.9%)	2,401 (68.6%)	591 (82.4%)	2,255 (77.6%)	
1	22 (7.8%)	65 (7.1%)	1,101 (31.4%)	126 (17.6%)	651 (22.4%)	
DM						<0.001
0	273 (96.8%)	856 (93.9%)	2,893 (82.6%)	615 (85.8%)	2,498 (86.0%)	
1	9 (3.2%)	56 (6.1%)	609 (17.4%)	102 (14.2%)	408 (14.0%)	
ALT, median (IQR)	20 (16, 24)	78 (47, 173.5)	17 (13, 22)	68 (46, 134)	42 (23, 82)	<0.001
AST, median (IQR)	22 (19, 25)	58 (37, 113.5)	22 (18, 26)	57 (37, 110)	34 (24, 66)	<0.001
TBIL, median (IQR)	10 (7, 13)	13 (9, 20)	10 (7, 14)	14 (10, 22)	12 (9, 20)	<0.001
ALB, median (IQR)	41.25 (36.2, 45.3)	40.5 (35.5, 45.8)	40.3 (36, 44.1)	38 (32.8,43)	39.9 (34.8,44.1)	<0.001
Plt, median (IQR)	209.5 (179, 252)	178 (135, 220)	201 (159, 245)	174 (124,216)	187 (141, 231)	<0.001
PT, median (IQR)	13.5 (12.9, 14.1)	13.7 (12.9,14.5)	13.9 (13.2, 15.1)	13.7 (13, 14.3)	13.6 (13,14.4)	<0.001
HBVDNA, median (IQR)	1.4E+8 (6.20E+7, 2.80E+8)	1.3E+7 (9.95E+, 1.05E+8)	290 (30,500)	7.5E+4 (8,900, 8.30E+5)	500 (44, 3,700)	<0.001

The baseline characteristics of patients in the immune-tolerant, HBeAg-positive immune active, inactive, HBeAg-negative immune-active, and GZ groups were shown in Table 1. Compared with the immune-tolerant and inactive groups, the GZ group had a higher proportion of male patients (*P* < 0.001). The median age of patients was higher in the GZ group compared with the immune-tolerant, HBeAg-positive immune active and HBeAg-negative immune-active groups (*P* < 0.001). Moreover, the median platelet count in the GZ group was higher than that in the immune-tolerant and inactive groups, and lower than that in the HBeAg-positive immune active and HBeAg-negative immune-active groups (*P* < 0.001). There were significant differences in total bilirubin, albumin, and PT among the 5 groups (*P* < 0.001).

### 3.2 IPTW-adjusted incidence of HCC in the entire cohort

**Figure 2:**
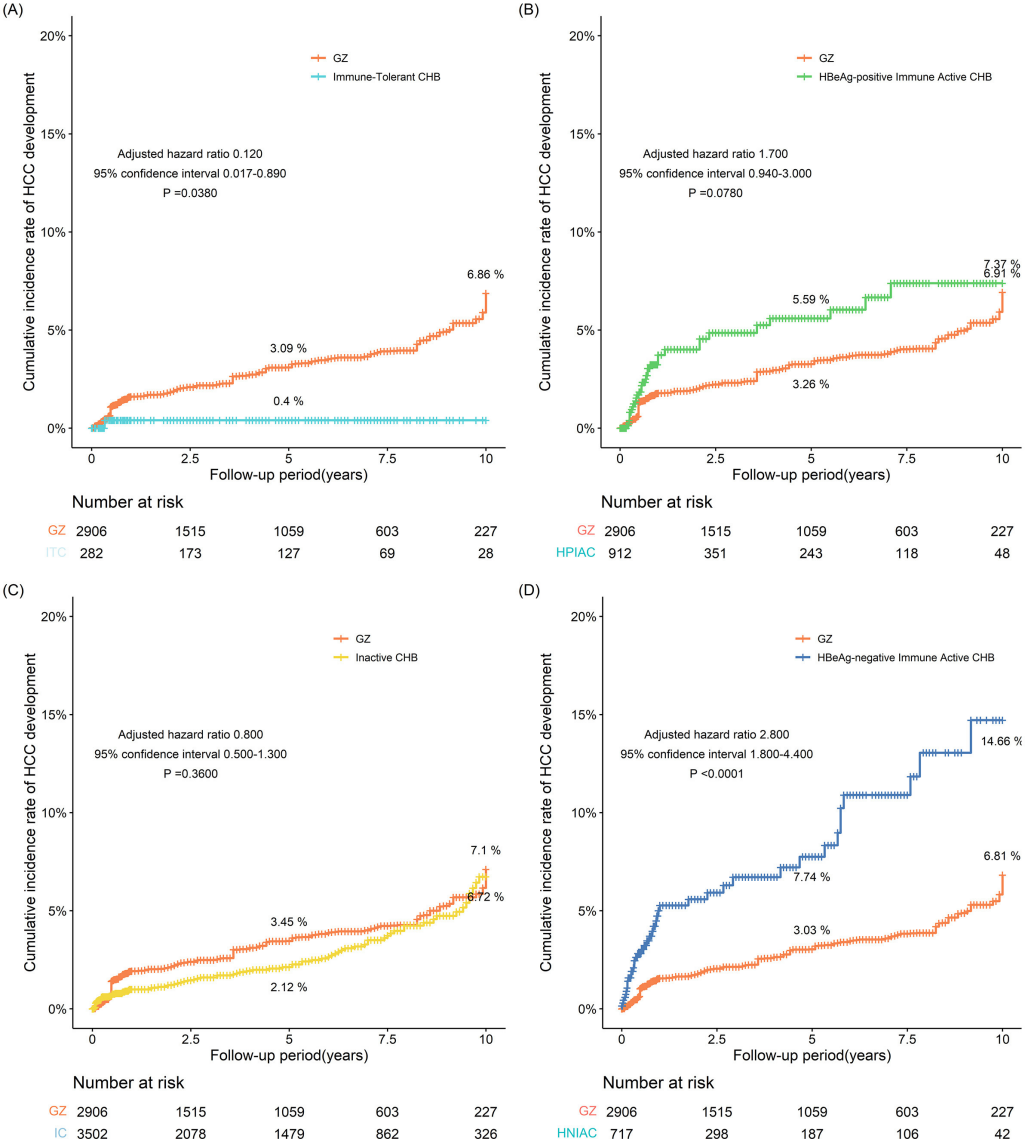
**(A–D)** The incidence of HCC adjusted for IPTW between the GZ group and the immune-tolerant, HBeAg-positive immune activity, inactive, and HBeAg-negative immune activation groups (IPTW, inverse probability of treatment weighting; GZ, gray-zone).

After IPTW, the 5- and 10-year incidences of HCC in the GZ group were 3.09% and 6.86%, respectively, while those in the immune-tolerant group were 0.400% and 0.362%, respectively, with statistically significant differences between the two groups (adjusted HR 0.120, 95% CI 0.017–0.890, *P* = 0.0380). After IPTW, the 5- and 10-year incidences of HCC were 3.26% and 6.91% in the GZ group and 5.59% and 7.37% in the immune-active group, respectively, and there was no statistically significant difference between the two groups (adjusted HR 1.700, 95% CI 0.940-3.000, *P* = 0.0780). After IPTW, the 5- and 10-year incidences of HCC were 3.45% and 7.10% in the GZ group, compared with 2.12% and 6.72% in the inactive group, and no statistically significant difference was found between the two groups (adjusted HR 0.800, 95% CI 0.500-1.300, *P* = 0.3600). After IPTW, the GZ group had 5- and 10-year incidences of HCC of 3.03% and 6.81%, respectively, while the reactivation group had 5- and 10-year incidences of HCC of 7.74% and 14.66%, respectively, with statistically significant differences between the two groups (adjusted HR 2.800, 95% CI 1.800–4.400, *P* < 0.0001). From the above results, it could be seen that after IPTW adjustment, the risk of HCC in the GZ group was higher than that in the immune-tolerant group, comparable to that in the immune-active group and the inactive group, and lower than that in the reactivation group (Figure 2).

### 3.3 Unadjusted incidence of HCC in GZ-A-GZ-D subgroups

According to different levels of HBeAg, ALT and HBV DNA (8, 9), GZ patients were divided into four categories: (1) Gray zone A (GZ-A): HBeAg positive, normal ALT level, HBV DNA ≤ 10^6^ IU/ml; (2) Gray zone B (GZ-B): HBeAg positive, ALT > ULN, HBV DNA ≤ 2 × 10^4^ IU/ml; (3) Gray zone C (GZ-C): HBeAg negative, normal ALT level, HBV DNA ≥ 2 × 10^3^ IU/ml; (4) Gray zone D (GZ-D): HBeAg negative, ALT > ULN, serum HBV DNA ≤ 2 × 10^3^ IU/ml. Among them, the ALT ULN was 25 U/L for females and 35U/L for males (1, 8).

There were 2,906 patients in the GZ group, including 340 in the GZ-A subgroup, 355 in the GZ-B subgroup, 705 in the GZ-C subgroup, and 1506 in the GZ-D subgroup. Compared with GZ-A and GZ-B subgroups, patients in the GZ-C and GZ-D subgroups were more elderly, had a higher incidence of hypertension and diabetes, and higher platelet values (*P* < 0.0001). There was no difference in albumin and PT among the four subgroups (Table 2).

**Table 2:** Baseline characteristic table for the four subgroups of GZ-A-GZ-D.

**Factor**	**GZ-A**	**GZ-B**	**GZ-C**	**GZ-D**	***P*-value**
N	340	355	705	1,506	
Sex	225 (66.2%)	258 (72.7%)	444 (63.0%)	1,051 (69.8%)	0.002
AGE, median (IQR)	47 (36, 58)	43 (32, 54)	52 (42, 61)	51 (41, 59)	<0.001
HT					<0.001
0	281 (82.6%)	315 (88.7%)	529(75.0%)	1,130 (75.0%)	
1	59 (17.4%)	40 (11.3%)	176 (25.0%)	376 (25.0%)	
DM					<0.001
0	311 (91.5%)	323 (91.0%)	608 (86.2%)	1,256 (83.4%)	
1	29 (8.5%)	32 (9.0%)	97 (13.8%)	250 (16.6%)	
ALT, median (IQR)	20 (15, 25)	93 (53, 235)	19 (15, 24)	60 (42, 114)	<0.001
AST, median (IQR)	23 (19, 29.5)	69 (39, 176)	23 (19, 27)	46 (32, 90)	<0.001
TBIL, median (IQR)	11 (8, 15)	17 (12, 46)	11 (8, 14)	13 (9, 22)	<0.001
ALB, median (IQR)	39.9(33.9,44.15)	39.9 (34.5, 44.2)	39.8 (35.5, 44)	39.9 (34.6, 44.2)	0.96
PLT, median (IQR)	176 (129, 229)	177 (121, 234)	199 (162, 236)	184 (138, 231)	<0.001
PT, median (IQR)	13.7 (13, 14.6)	13.6 (12.8, 14.6)	13.5 (13, 14.2)	13.6 (13, 14.5)	0.29
HBVDNA, median (IQR)	500 (30, 7,700)	520 (120, 2,700)	8,000 (4,100, 26,000)	440 (30, 500)	<0.001

**Figure 3:**
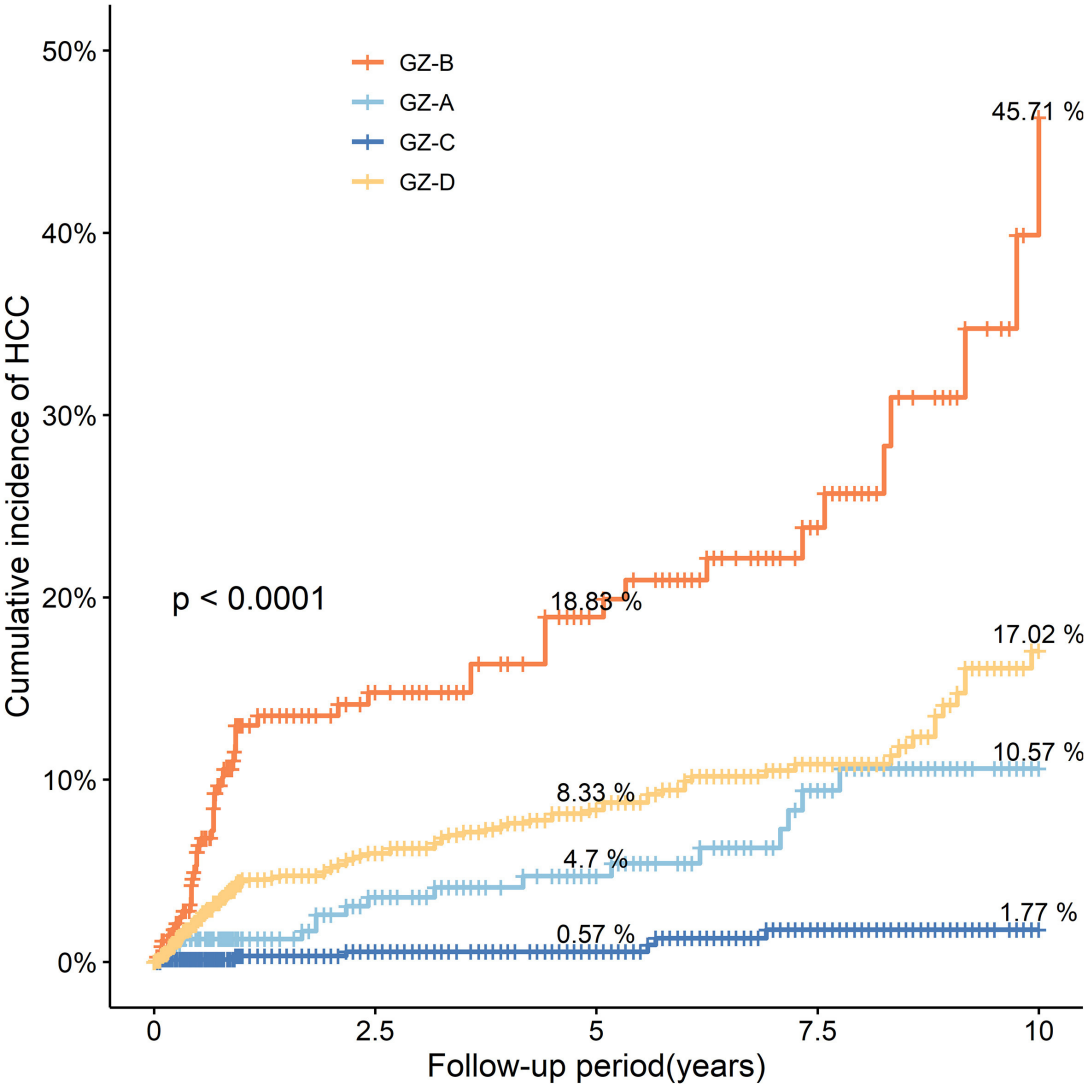
The probability and risk of developing HCC in the four subgroups GZ-A-GZ-D without IPTW adjustment (IPTW, inverse probability of treatment weighting; HCC, hepatocellular carcinoma; GZ, gray-zone).

The incidence of HCC in the GZ-A subgroup after 5 years of follow-up was 4.70%, and that after 10 years of follow-up was 10.57%. The incidence of HCC in the GZ-B subgroup after 5 and 10 years of follow-up was 18.83% and 45.71%, respectively. The incidence of HCC in the GZ-C subgroup was 0.57% after 5 years of follow-up and 1.77% after 10 years of follow-up. Iin the GZ-D subgroup, the incidence of HCC after 5 years of follow-up was 8.33% and that after 10 years of follow-up was 17.02%. The GZ-B subgroup had the highest incidence of HCC, and there was a significant difference (*P* < 0.001) among the four groups (Figure 3).

### 3.4 Incidence of HCC in the GZ-A-GZ-D subgroups after IPTW adjustment

**Figure 4:**
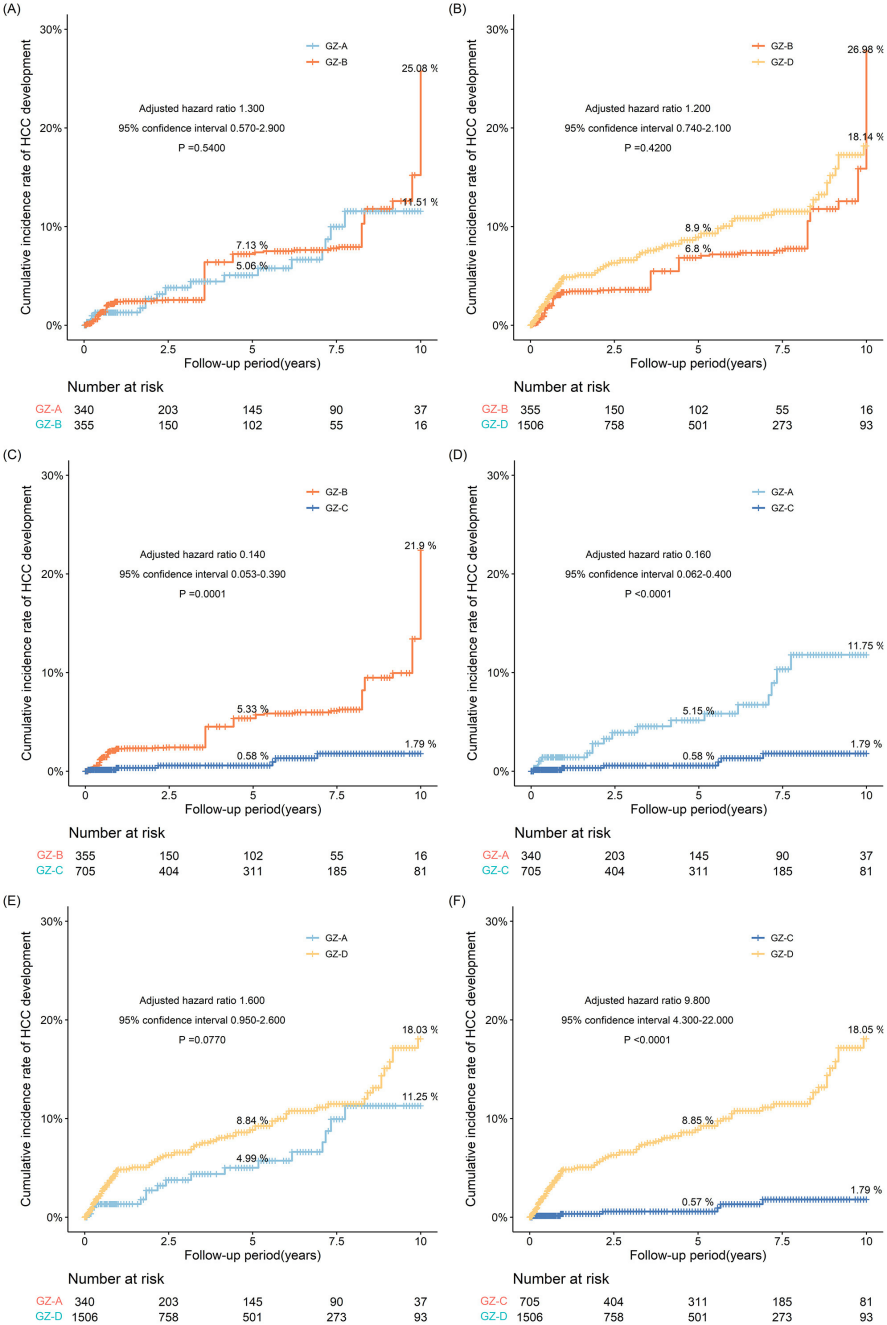
**(A–F)** The risk probability of developing HCC in GZ-A-GZ-D subgroups after IPTW adjustment.

After IPTW adjustment, the incidence of HCC in the GZ-B subgroup was similar to that in the GZ-A and GZ-D subgroups, and significantly increased compared to the GZ-C subgroup (*P* < 0.001) (Figures 4A–C). Considering that the GZ-B subgroup had a relatively higher incidence of HCC among the four subgroups, the incidence of HCC in the GZ-B subgroup was compared with that in the immune-tolerant, HBeAg-positive immune active, inactive, and HBeAg-negative immune-active groups.

After IPTW adjustment, the 5- and 10-year incidences in the GZ-D subgroup were 8.9% and 18.14%, respectively, and the 5- and 10-year incidence in the GZ-B subgroup were 6.8% and 26.98%, respectively. However, there was no statistical difference between the two subgroups (*P* = 0.420). After IPTW adjustment, the 5- and 10-year incidences were 8.84% and 18.03% in the GZ-D subgroup and 4.99% and 11.25% in the GZ-A subgroup, respectively. However, after comparing the two subgroups as a whole, no statistical difference was observed between the two subgroups (*P* = 0.0770). After IPTW adjustment, the 5- and 10-year incidences in the GZ-D subgroup were higher than those in the GZ-C subgroup. After comparing the two subgroups, it was found that the incidence in the GZ-D subgroup was significantly higher than that in the GZ-C subgroup, and there was a statistical difference (*P* < 0.0001) (Figures 4D–F).

**Figure 5:**
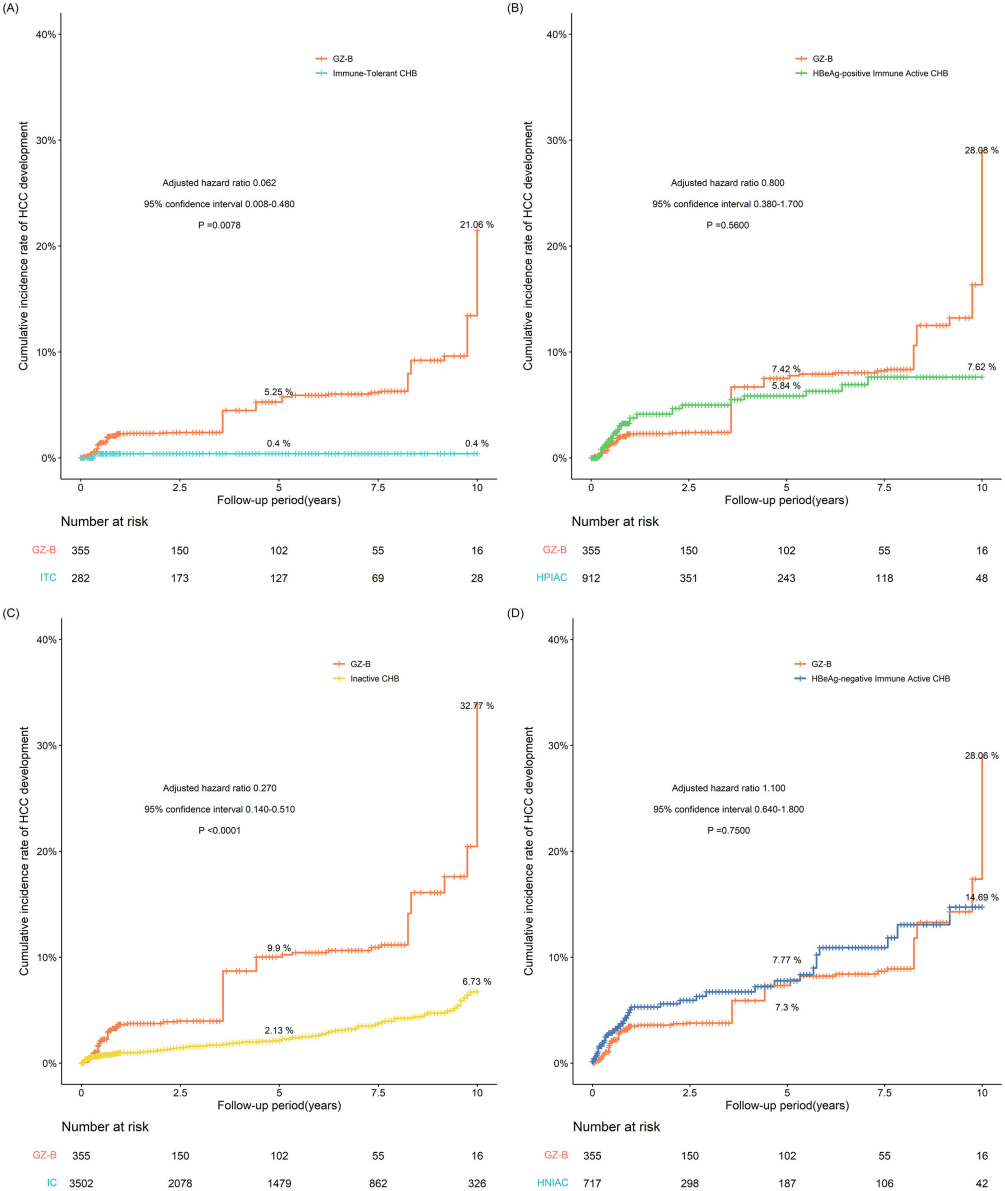
**(A–D)** The probability of developing HCC after IPTW adjustment between GZ-B subgroup and immune tolerant group, HBeAg-positive immune activity, inactive group, and HBeAg-negative immune activation group (IPTW, inverse probability of treatment weighting; GZ, gray-zone).

Due to the highest risk of HCC in the GZ-B subgroup, a comparison was made between the GZ-B subgroup and the immune-tolerant, HBeAg-positive immune active, inactive, and HBeAg-negative immune-active groups. The results showed that the GZ-B subgroup had a higher risk of HCC compared to the immune active, HBeAg-positive immune active, inactive, and HBeAg-negative immune-active groups. After IPTW adjustment, the results indicated that the probability of developing HCC in the GZ-B subgroup was comparable to that in the HBeAg-positive immune active and HBeAg-negative immune-active groups, and higher than that in the immune-tolerant group (adjusted HR 0.062, 95%CI 0.008–0.480, *P* = 0.0078) and the inactive group (adjusted HR 0.270, 95%CI 0.140–0.510, *P* < 0.0001) (Figure 5).

## 4 Discussion

In this study, the incidence of HCC in the GZ group was compared with that in the immune-tolerant, HBeAg-positive immune active, inactive, and HBeAg-negative immune-active groups. This observational study indicated that after IPTW adjustment, the incidence of HCC in the GZ group was similar to that in immune-tolerant, HBeAg-positive immune active and inactive groups, but lower than that in the reactivation group.

The long-term immune response of the host to hepatitis B virus could cause chronic liver inflammation, leading to a continuous circulation of hepatocyte death and regeneration. As the fibrosis stage progressed, the proportion of patients with significant inflammatory activity increased significantly in those with ALT > 2 × ULN (6, 8). The proportion of CHB patients with significant inflammation increased with the stage of liver fibrosis, regardless of HBeAg status, HBV DNA levels, or age category (14). Subsequently, the risk of cirrhosis and HCC would significantly increase (15). High serum HBV DNA levels in CHB patients were associated with a high risk of HCC (11). In CHB patients with detectable HBV DNA, ALT > 2 × ULN and the presence of significant fibrosis were independent risk factors for significant inflammation (16). The incidence of HCC in the active and HBeAg-negative immune-active groups was higher than that in the GZ group, but the incidence of HCC in the HBeAg-negative immune-active group had decreased significantly after anti-hepatitis B virus treatment.

Lee et al. studied untreated non-cirrhotic patients with HBeAg positive CHB in the immune-tolerant group. Thirteen studies were included, with an overall median follow-up period of 62.4 months. The 5- and 10-year incidences of HCC in the immune-tolerant group were 1.1% and 2.7%, respectively (17), which were similar to our results. Seung In Seo et al. found that during an average follow-up of 63 months, the incidence of HCC in the inactive group was 4.5% (18). Another analysis of 7,977 untreated patients in the inactive group found that the annual incidence of HCC was about 3.8% (19), similar to our results. From our study, it could be seen that after IPTW adjustment, the probability of developing HCC in the GZ group was similar to that in the immune-tolerant, HBeAg-positive immune active, and inactive groups, but lower than that in the HBeAg-negative immune-active group. Huang DQ et al. found that GZ patients had a higher 10-year cumulative incidence of HCC (4.6% vs. 0.5%; *P* < 0.0001) and an adjusted HCC risk ratio of 14.1 (*P* = 0.03) compared to inactive patients (7). Patients in the GZ group had a higher incidence of HCC, the liver inflammation (≥G2) and significant liver fibrosis (≥S2) of whom were less severe than those in the HBeAg-positive immune active group, but more severe than those in the immune-tolerant and inactive groups (10). About 72.7% of CHB patients in GZ stage suffered from SHD, i.e., liver inflammation ≥G2 and/or liver fibrosis ≥S2 (10).

In addition, the GZ group could be divided into four subgroups. Our findings suggested that the GZ-B subgroup had the highest risk of HCC. The IPTW-adjusted analysis showed that the probability of developing HCC in the GZ-B subgroup was similar to that in the HBeAg-positive immune active and HBeAg-negative immune-active groups, but higher than that in the immune-tolerant and inactive groups. Among GZ patients, GZ-B patients had the highest proportion of SHD (100.0%), followed by GZ-A (84.0%), GZ-D (69.9%), and GZ-C (67.0%) (10). On the other hand, it was known that the degree of liver injury was closely associated with the risk of HCC in CHB patients (20). The SHD in the pathology of GZ-B subgroup was consistent with the risk trend of HCC in 10-year follow-up of our patients. The GZ-D subgroup still had a high incidence of HCC, which should be brought to the attention of clinical physicians.

According to APASL, AASLD, and EASL criteria, the proportion of patients who developed HCC outside treatment recommendations was 64.0%, 46.0%, and 33.5%, respectively (1–3). The 5-year cumulative incidence of HCC in cirrhosis patients with low-level viremia was 13.9%, and that in CHB patients with elevated HBV DNA level and slightly elevated ALT level was 6.1%−7.3% (21). More than 90% of HbeAg-positive GZ CHB patients exhibited SHD (10). Most GZ CHB patients were HbeAg-negative, with nearly 70% exhibiting SHD (10). Kumar et al. found that approximately 50% of HbeAg-negative patients with normal ALT had SHD (22). In a study by Lai et al., 37% of CHB patients with normal ALT levels had significant fibrosis or inflammation (23). If the biopsy specimen showed moderate or severe inflammation (A2 or A3) or obvious fibrosis (≥F2), anti-hepatitis B virus therapy was recommended (1, 10). More than 80% of GZ-A and GZ-B CHB patients developed SHD, so antiviral therapy was recommended (10). GZ-C CHB patients with PT ≥ 12.6s and GZ-D CHB patients with PT ≥ 12.4 s were highly likely to develop SHD, therefore antiviral therapy was recommended (10).

This study has several limitations. Firstly, there might be selection bias and confusion in the results of this retrospective observational study. Therefore, IPTW was used in this study to adjust the results. However, unmeasured confounding factors cannot be fully explained, and further prospective double-blind controlled clinical trials are needed to validate these results. Nevertheless, as prospective clinical trials involve lots of time and effort, retrospective observational studies are still worth learning from. Secondly, commonly used clinical and radiological criteria were used in this study to diagnose HCC. After being diagnosed with HCC, some patients received interventional therapy without definite pathological results. Finally, the results, although still credible and reliable to a certain extent due to the large amount of data in our center and a follow-up period of over 10 years, as single-center study data, need to be further validated with data from other centers.

In summary, the probability of developing HCC in the GZ group was similar to that in the immune-tolerant, HBeAg-positive immune active, and inactive groups. The probability of developing HCC in the GZ-B subgroup was similar to that of the HBeAg-positive immune active and HBeAg-negative immune-active groups. The GZ group and GZ-B subgroup had a higher risk of HCC. Anti-hepatitis B virus therapy should be considered as early as possible for patients in the GZ group, especially in the GZ-B subgroup.

## Data Availability

The raw data supporting the conclusions of this article will be made available by the authors, without undue reservation.
